# Evaluating the modified spinal cord injury secondary conditions scale (SCI-SCS) combining severity and mortality-based weights

**DOI:** 10.1038/s41393-026-01167-4

**Published:** 2026-01-16

**Authors:** Ana Oña, Vegard Strøm, Carla Sabariego, Armin Gemperli, Diana Pacheco Barzallo

**Affiliations:** 1 Swiss Paraplegic Research, Guido A. Zäch Institute, Nottwil, Switzerland; 2 Faculty of Health Sciences and Medicine, University of Lucerne, Lucerne, Switzerland; 3 Sunnaas Rehabilitation Hospital, Nesoddtangen, Norway; 4 Center for Rehabilitation in Global Health Systems, University of Lucerne, Lucerne, Switzerland; 5 Center of Primary and Community Care, University of Lucerne, Lucerne, Switzerland

**Keywords:** Disability, Diseases

## Abstract

**Study design:**

Cross-sectional study

**Objective:**

To evaluate alternative scoring approaches for the modified Spinal Cord Injury Secondary Conditions Scale (SCI-SCS) using severity- and mortality-based weights, and to examine their associations with functioning and self-reported health in individuals with SCI.

**Setting:**

Community

**Methods:**

We analyzed data from 10,347 participants in the International Spinal Cord Injury Survey (InSCI). Eight scoring approaches were constructed from 14 secondary health conditions and a depression item, varying by severity coding and by whether mortality weights were applied equally or condition-specifically. Associations with functioning (ICF-based composite score) and self-reported health were assessed using Pearson and Spearman correlations, with additional country-level analyses to explore variability.

**Results:**

All scoring approaches were negatively correlated with both outcomes, indicating that higher secondary health condition burden was associated with worse functioning and poorer health. The score combining the modified SCI-SCS with condition-specific mortality weights showed the highest correlations, though differences from the unweighted score were small. Both scores demonstrated moderate associations with the outcomes. Country-level analyses revealed variability, partly related to sample size, but overall patterns were consistent.

**Conclusion:**

The modified SCI-SCS demonstrated moderate and robust associations with functioning and self-reported health, supporting its use as a pragmatic proxy of overall health status in individuals with SCI. Weighting by mortality risks yielded only marginal gains, suggesting that the unweighted score remains a suitable option for research and practice. These findings advance the use of self-reported measures to capture health burden in SCI and encourage further validation with independent outcomes and across diverse contexts.

## Introduction

Spinal Cord Injury (SCI) is a chronic health condition characterized by profound and life-disrupting consequences [1]. A major challenge of spinal cord injury (SCI) is the development of secondary health conditions, which often arise after injury and intensify with aging [2]. The most common conditions are pain, breathing problems, and infections, which directly affect well-being [3]. At a broader population level, data on secondary health conditions are essential to identify risk groups, assess the impact on health outcomes, plan and deliver services, and shape evidence-based health policies [4].

Comorbidity indices provide a way to quantify the presence of different health conditions within an individual, and several methods have been developed for this purpose. These indices are widely applied in health research and practice, for example, to predict mortality, estimate hospital load, or serve as case-mix adjustment factors in hospital comparisons [5]. Among them, the Charlson Comorbidity Index (CCI) is the most commonly used, as it accounts for both the number and severity of a person’s comorbidities [6]. To extend such approaches to patient-reported information, self-reported comorbidity scales have been developed to capture insights directly from survey or questionnaire data [7]. In line with this, indices modeled after the CCI have been adapted [8] to provide a broader understanding of the health status and healthcare [9] of specific populations [10].

Although the CCI is a valuable benchmark and can often be derived from routinely collected clinical data, such data are not always available, a limitation that is particularly problematic in the context of spinal cord injury (SCI). To capture the extent of secondary health conditions in this population, alternative measures have therefore been developed. One of the most prominent is the SCI Secondary Conditions Scale (SCI-SCS), a self-reported instrument that has been widely used both in clinical practice and research to document the burden of secondary health conditions among persons with SCI [11, 12]. Despite its extensive use, the SCI-SCS has faced criticism due to the limited psychometric evidence regarding its validity, reliability, and applicability across diverse contexts [13]. Moreover, while different scoring approaches have been applied, it remains unclear how well scores capture the burden of secondary health conditions on health outcomes. Nevertheless, in the absence of better alternatives, both researchers and clinicians continue to rely on the SCI-SCS to describe morbidity and its burden. The aim of this paper is to address this gap by testing alternative scoring approaches for the modified SCI-SCS, combining severity and mortality weights, and examining their association with relevant SCI outcomes.

## Methods

### Data

The analysis used data from the International Spinal Cord Injury Survey (InSCI), which is the first survey on community-dwelling people with an SCI across all six WHO regions [14]. InSCI collected comparable information on the living situation of people with an SCI across 22 countries [15, 16]. The survey questionnaire was structured using the International Classification of Functioning, Disability and Health (ICF) as a reference framework, facilitating cross-country comparisons. The comprehensive survey encompassed 11 sections, including information on personal-psychological factors, health-related dimensions, and environmental determinants [17].

### Sample

The InSCI Survey was conducted between 2017 and 2019 across 22 countries and included data from 12,591 participants worldwide [18]. Eligible participants were adults aged 18 years or older with either traumatic or non-traumatic SCI, who were able to complete the questionnaire and provided informed consent [17]. Neurological characteristics (paraplegia/tetraplegia; complete/incomplete) were self-reported and described in line with the ASIA/ISCoS International Standards for Neurological Classification of Spinal Cord Injury (ISNCSCI). To ensure sufficient statistical power, a minimum target sample size of 200 participants per country was established [17, 19]. Individuals with congenital etiologies such as spina bifida, as well as those with neurodegenerative disorders or peripheral nerve injuries (e.g., Guillain-Barré syndrome), were excluded from the study [17–19].

### Score construction

#### Score bases

The SCI Secondary Conditions Scale (SCI-SCS) was originally developed to capture the prevalence and burden of secondary health conditions in people with SCI and has been widely applied in both clinical and research contexts, particularly for cross-sample comparisons and for examining associations with health and well-being outcomes [7, 12]. For the InSCI survey, the instrument was adapted to address limitations in the original format. Specifically, the response options of the SCI-SCS, which mixed severity and frequency and proved problematic both for respondents and for translation, were replaced with a uniform 5-point scale derived from the WHO Model Disability Survey. Participants were asked to report how much of a problem each condition posed in the last three months, ranging from *1 = no problem* to *5 = extreme problem*, with only the endpoints labeled [19].

In the InSCI version, 14 specific physical health conditions were included, covering a broad spectrum of issues such as respiratory and circulatory problems, bowel and bladder dysfunction, spasticity, pressure ulcers, sensation-loss injuries, autonomic dysreflexia, sleep disturbances, sexual dysfunction, contractures, urinary tract infections, postural hypotension, and pain. Compared to the original 16-item SCI-SCS, three notable changes were made: (1) diabetes mellitus was excluded and captured instead by an open-ended question on additional health conditions, (2) joint and muscle pain was replaced with a broader item on pain, and (3) heterotopic ossification, being relatively uncommon and difficult to capture through self-report, was removed. To harmonize the structure and ensure coverage of relevant domains, two new items were added on sleep functions and pain interference with daily activities, aligning with ICF categories [17–19].

An additional modification concerned the response scales. While most countries in the InSCI study used the modified 5-point problem-severity scale, Switzerland retained the original 4-point ordinal scale but applied it to the 14 items. This version combines the frequency and intensity of health problems using the categories: (1) not experienced or insignificant problem, (2) mild or infrequent problem, (3) moderate or occasional problem, and (4) significant or chronic problem [20, 21]. To ensure comparability with the other countries, Swiss data were excluded from the main computations but analyzed separately to validate the results (see Appendix).

Recognizing the high prevalence of mental health problems in SCI populations [2], a question on depressive symptoms was also included, derived from the SF-36v2 Mental Health Subscale. Participants indicated how often they felt “downhearted and depressed” during the last four weeks, using a 5-point scale ranging from *1 = all of the time* to *5 = none of the time* [17]. Together, these 15 items from the modified SCI-SCS score that we are using in this paper.

#### Score combinations

To develop the scoring approaches, we tested several versions using a combination of severity and mortality weights for the reported health conditions. First, we recoded the scale from 1–5 to 0–4 to ensure no weight was assigned when participants reported no problem. We also recoded the depression options to have the same order than the secondary health conditions.

##### Severity of the health problems

We used four distinct approaches: (1) the original responses in the questionnaire, which are *0 for no problem, 1 for insignificant problems, 2 for mild, 3 for moderate and 4 for severe problems reported*, (2) dichotomized as 1 for *severe and moderate problem and 0 otherwise*, (3) dichotomized as 1 *for reporting a problem* and 0 otherwise, and (4) grouped into *three severity groups* as 3 attributed to cases reporting severe or moderate problems, 1 to mild and insignificant problems, and 0 otherwise.

##### Mortality weights

To reflect the varying impacts of secondary health conditions on overall health and survival, we applied weights based on their associated mortality risk. Higher weights were assigned to conditions with a well-documented association with increased mortality, particularly respiratory and circulatory problems, given their direct impact on vital functions. Conditions with recognized but less immediately life-threatening consequences were assigned intermediate or lower weights, reflecting clinical evidence and previous research on their contribution to morbidity and long-term health complications.

Two approaches to weighting were applied: first, an unweighted model in which all conditions were treated equally, and second, a disease-based weighting scheme tailored to the SCI population. In this scheme, the highest mortality risk weight (3) was allocated to respiratory and circulatory problems; an intermediate weight (2) to bowel and bladder dysfunction, muscle spasms, pressure ulcers, sensation-loss injuries, autonomic dysreflexia, urinary tract infections, and depression; and the lowest weight (1) to sleep problems, sexual dysfunction, contractures, postural hypotension, and pain [22]. A detailed overview of the SCI-specific mortality weights, along with brief justifications and supporting references, is provided in Table A1 (Appendix).

Table 1 presents the different scoring approaches, including the specific combinations of weights and severity levels applied. In total, we built 8 scores, where the score 1 was the modified SCI-SCS scale. For all approaches, the minimum possible score was *0*, indicating the absence of health problems. The maximum score varied depending on the weighting method, with the highest attainable score being *108 points*. Formally, the scoring procedure can be described as follows:

$${{Score}}_{i}={\sum }_{j=1}^{15}\left\{{\beta }_{{ij}}* {\omega }_{j}\right\},$$

where ${{Score}}_{{i}}$ is the sum of the 15 *(j)* health problems for the individual *(i)* according to the severity ${\beta }_{{ij}}$ times the mortality risk ${\omega }_{j}$.

**Table 1:** Score combinations.

Scores	How the severity reported was used?					Which weights were used?		Score ranges	
	No problem	Insignificant	Mild	Moderate	Severe	Equal weights	Mortality weights	Mean	Max
Score 1: modified SCI-SCS	0	1	2	3	4	yes		16.8	56
Score 2	0	1	2	3	4		yes	30.4	108
Score 3	0	0	0	1	1	yes		5.0	27
Score 4	0	0	0	1	1		yes	3.0	15
Score 5	0	1	1	1	1	yes		8.2	15
Score 6	0	1	1	1	1		yes	14.0	27
Score 7	0	1	1	3	3	yes		14.1	44
Score 8	0	1	1	3	3		yes	23.7	79

Table 1 shows the combinations of the scores with the 2 variables severity and mortality weights. Severity reported: (1) original values reported, (2) dichotomized as 1 for severe and moderate problem and 0 otherwise, (3) dichotomized as 1 for reporting a problem and 0 otherwise, and (4) grouped into three severity groups as 2 attributed to cases reporting severe or moderate problems, 1 to mild and insignificant problems, and 0 otherwise. Mortality weights: a) equal weights or no weights and b) mortality weights.

### Comorbidity score validation

To examine the concurrent validity of the new scoring approaches against the modified SCI-SCS overall score, we correlated both with two external measures: functioning (as defined by the ICF) [23], and self-reported health status, a well-established predictor of mortality [24]. Both measures were collected as part of the InSCI survey [14].

**Functioning** was assessed using a composite score that reflects daily activities, mobility, and participation in social and economic life, covering both physical and psychological aspects of health. The score is based on 22 items, 7 of which overlap with the modified SCI-SCS (secondary health conditions) plus one depression item. The items cover domains such as self-care, mobility, community involvement, work and leisure, and emotional well-being, in line with the ICF framework [23]. Scores were combined to produce an overall functioning score, which ranges from 0 (worst level of functioning) to 100 (best level of functioning) [23]. **Self-reported health status** was measured with a single-item question in which participants rated their overall health on a scale from *poor* to *excellent* [17, 18].

For the functioning score, correlations were calculated using Pearson’s correlation coefficient. For self-reported health status, a Spearman correlation was applied, since this measure is assessed on an ordinal five-point Likert scale (*poor, fair, good, very good, excellent*). To compare the strength of the correlation coefficients, we conducted Steiger’s test. All statistical analyses were performed using R, version 4.2.2.

## Results

### Descriptive statistics

The final dataset comprised 10,347 participants, with males accounting for approximately 73% of the total. The average age of the participants was 50.4 years old, with a range spanning from a minimum age of 18 (per study design) to a maximum of 96 years. On average, participants had lived 14 years with the injury, and close to 61% of participants exhibited incomplete lesions. Approximately 61% of participants had paraplegia. The mean of the functioning score was 49.8 (SD 15.6), and 45.4% of the participants reported fair or poor health. About 40% of the participants were concentrated in Australia, China, or Germany (Table 2).

**Table 2:** Descriptive statistics, n = 10347.

	Overall
**Sex**	
Male	7593 (73.4%)
Female	2741 (26.5%)
Missing	13 (0.1%)
**Age group**	
18–30	1137 (11.0%)
30–40	1732 (16.7%)
40–50	2239 (21.6%)
50–60	2394 (23.1%)
60–70	1785 (17.3%)
>70	1005 (9.7%)
Missing	55 (0.5%)
**Years since the injury**	
Mean (SD)	14.1 (11.4)
Median [Min, Max]	10.5 [0.5, 74.5]
Missing	166 (1.6%)
**Degree**	
Complete	3911 (37.8%)
Incomplete	6325 (61.1%)
Missing	111 (1.1%)
**Type**	
Paraplegia	6286 (60.8%)
Tetraplegia	3849 (37.2%)
Missing	212 (2.0%)
**Functioning score**	
Mean (SD)	49.8 (15.6)
Median [Min, Max]	49.0 [0, 100]
**Self-reported health**	
Poor	1316 (12.7%)
Fair	3387 (32.7%)
Good	3930 (38.0%)
Very good	1269 (12.3%)
Excellent	266 (2.6%)
Missing	179 (1.7%)
**Country**	
Australia	1374 (13.3%)
Brazil	199 (1.9%)
China	1354 (13.1%)
France	380 (3.7%)
Germany	1418 (13.7%)
Greece	197 (1.9%)
Indonesia	194 (1.9%)
Italy	197 (1.9%)
Japan	279 (2.7%)
Lithuania	215 (2.1%)
Malaysia	283 (2.7%)
Morocco	385 (3.7%)
Netherlands	229 (2.2%)
Norway	579 (5.6%)
Poland	918 (8.9%)
Romania	213 (2.1%)
South Africa	197 (1.9%)
South Korea	851 (8.2%)
Spain	389 (3.8%)
Thailand	311 (3.0%)
USA	185 (1.8%)

Table 3 outlines the health conditions reported by participants, categorized by severity. Among the health conditions, sexual problems emerged as the most frequently reported as *severe* (29.6%), followed by pain (16.2%), bladder problems (13.2%), spasticity (13.0%), and contractures (12.6%). In contrast, respiratory problems, for instance, were reported as *no problem* for 69.8% of the participants, followed by injury caused by loss of sensation (68.3%) and pressure ulcers (63.7%).

**Table 3:** Descriptive statistics of the health problems.

Health problems	Severity reported					
	No problem	Insignificant	Mild	Moderate	Severe	Missing
Sleep problems	3642 (35.2%)	1960 (18.9%)	2319 (22.4%)	1435 (13.9%)	795 (7.7%)	196 (1.9%)
Bowel problems	2969 (28.7%)	2214 (21.4%)	2171 (21.0%)	1510 (14.6%)	1261 (12.2%)	222 (2.1%)
Urinary problems	4862 (47.0%)	1677 (16.2%)	1542 (14.9%)	1070 (10.3%)	910 (8.8%)	286 (2.8%)
Bladder problems	3868 (37.4%)	1860 (18.0%)	1644 (15.9%)	1321 (12.8%)	1370 (13.2%)	284 (2.7%)
Sexual problems	2799 (27.1%)	979 (9.5%)	1505 (14.5%)	1424 (13.8%)	3067 (29.6%)	573 (5.5%)
Contractures	3218 (31.1%)	1873 (18.1%)	2067 (20.0%)	1557 (15.0%)	1308 (12.6%)	324 (3.1%)
Spasticity	2716 (26.2%)	2053 (19.8%)	2234 (21.6%)	1781 (17.2%)	1347 (13.0%)	216 (2.1%)
Pressure ulcers	6591 (63.7%)	1274 (12.3%)	852 (8.2%)	609 (5.9%)	764 (7.4%)	257 (2.5%)
Respiratory problems	7218 (69.8%)	1342 (13.0%)	844 (8.2%)	437 (4.2%)	243 (2.3%)	263 (2.5%)
Injury loss	7062 (68.3%)	1191 (11.5%)	796 (7.7%)	550 (5.3%)	458 (4.4%)	290 (2.8%)
Circulatory problems	4797 (46.4%)	1950 (18.8%)	1744 (16.9%)	1040 (10.1%)	563 (5.4%)	253 (2.4%)
Dysreflexia	6114 (59.1%)	1731 (16.7%)	1188 (11.5%)	668 (6.5%)	373 (3.6%)	273 (2.6%)
Hypotension	6343 (61.3%)	1807 (17.5%)	1095 (10.6%)	508 (4.9%)	289 (2.8%)	305 (2.9%)
Pain	2330 (22.5%)	1972 (19.1%)	2264 (21.9%)	1879 (18.2%)	1675 (16.2%)	227 (2.2%)
	**None of the time**	**A little of the time**	**Some of the time**	**Most of the time**	**All the time**	**Missing**
Depression	2161 (20.9%)	3795 (36.7%)	2956 (28.6%)	994 (9.6%)	284 (2.7%)	157 (1.5%)

### Score validation

**Fig. 1:**
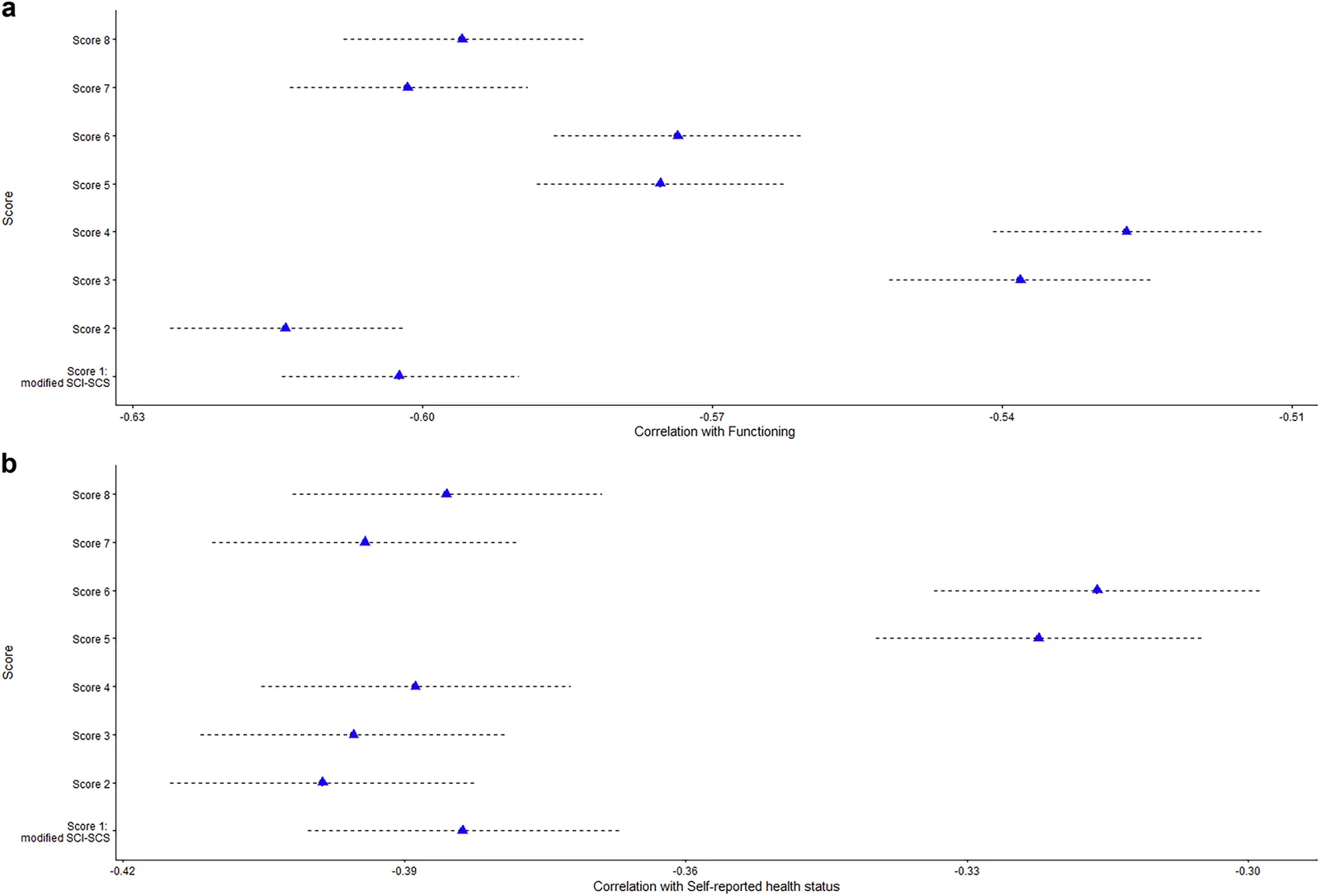
Validation of the scores using two health outcomes. **a** Correlation coefficients with 95% confidence intervals between the scores and functioning*. *: We applied a Steiger’s test to test the differences between the correlation coefficients between Score 1 and Score 2, using average correlations z = 8.8747, *p* value = 0.0000 the null hypothesis rejected, meaning that even though Score 1 and Score 2 are similar, the relationship with functioning differ significantly. **b** Correlation coefficients with 95% confidence intervals between the scores and self-reported health status*. *: We applied a Steiger’s test to test the differences between the correlation coefficients between Score 1 and Score 2, using average correlations z = 8.9126 *p* value = 0.0000 the null hypothesis rejected, meaning that even though Score 1 and Score 2 are similar, the relationship with self-reported health status differ significantly.

Figures 1a and 1b illustrate the validation of the scores using two health outcomes: functioning and self-reported health status. The scores were negatively correlated with the two outcomes, meaning that a higher score is associated with worse functioning and health status.

Among the scoring approaches, the combined modified SCI-SCS and mortality-weighted score (Score 2) demonstrated the strongest correlations with both outcomes, followed closely by the unweighted modified SCI-SCS score (Score 1). In contrast, Scores 3 and 4, which only considered problems reported at the most extreme severity, performed the poorest in relation to functioning. For self-reported health status, the weakest associations were observed for Scores 5 and 6, which relied on a dichotomous (presence/absence) coding of health problems.

Given the close similarity between Score 1 and Score 2, we used Steiger’s test to examine whether the differences in their correlation coefficients were statistically significant. The results indicated that, for both indices, the strength of the relationship with the health outcomes differed significantly.

**Fig. 2:**
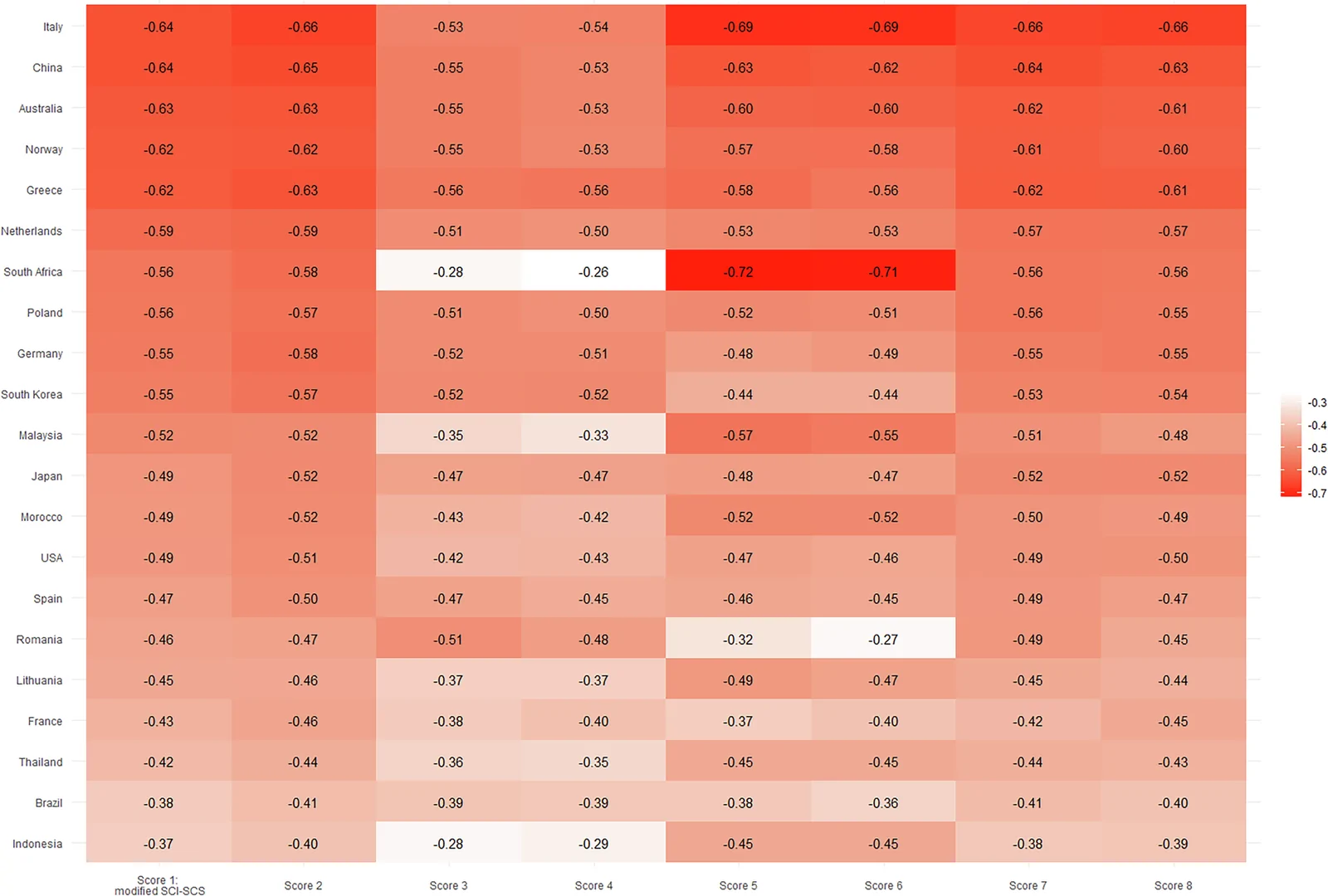
Pearson correlation between the scores and functioning by country.

**Fig. 3:**
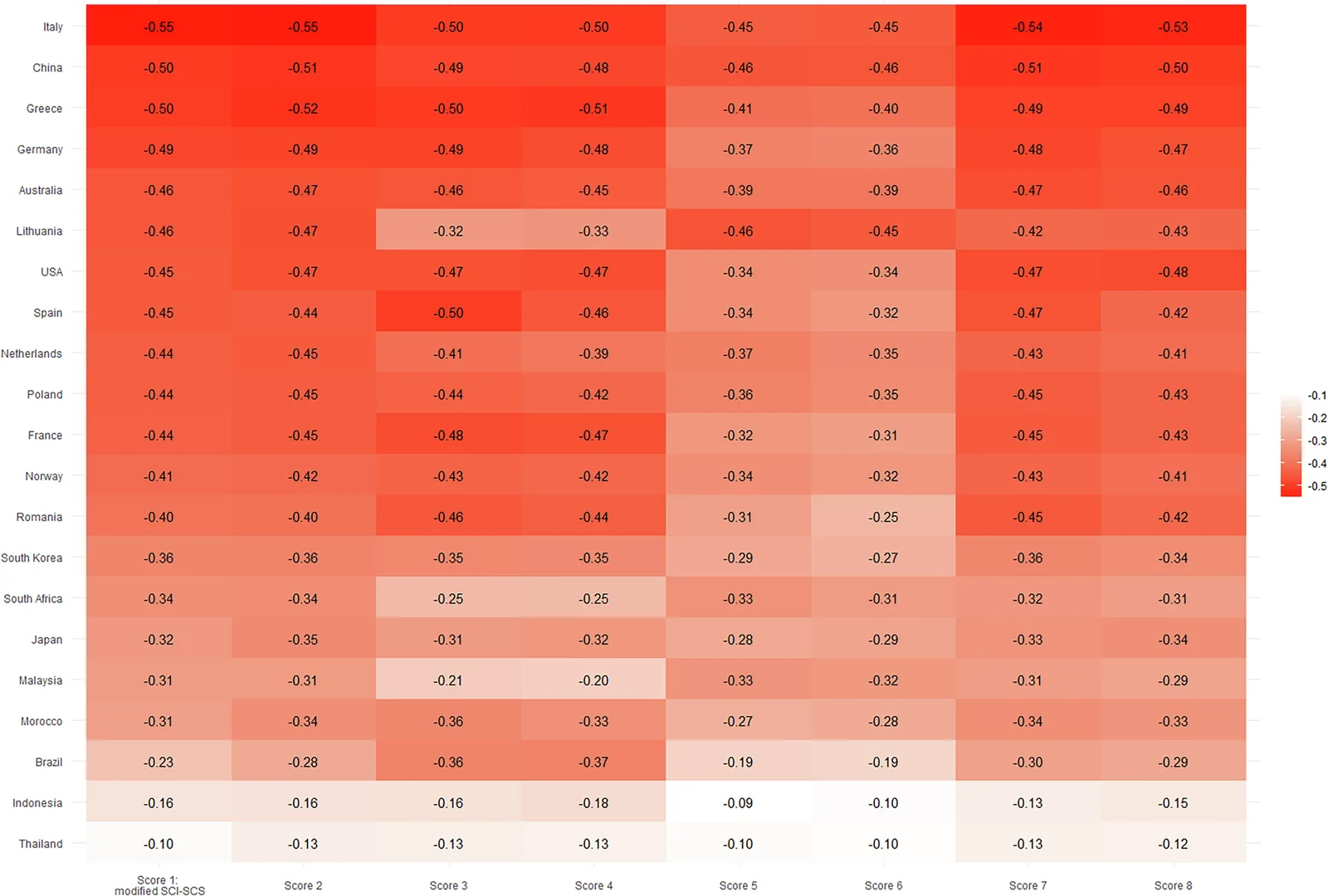
Spearman correlation between scores and self-reported health status by country.

Figures 2 and 3 present the correlations of the different scores with functioning and self-reported health, respectively, across countries using heat maps, where darker colors indicate stronger correlations. Across most countries, Score 2 consistently showed the strongest correlations, followed closely by the modified SCI-SCS score. However, the strength of associations varied by country, with more stable patterns observed in countries with larger sample sizes—suggesting that sample size may partly account for the variability.

The Swiss InSCI sample, which applied the original four-point SCI-SCS response categories, was analyzed separately as a validation sample. As shown in the Appendix, the Swiss results revealed patterns of association similar to those observed in the main analyses, supporting the robustness of our findings across different option’s formats.

## Discussion

This study explored alternative approaches to constructing weighted scores based on the modified SCI-SCS, using a large cross-national dataset for validation. We developed seven scoring variants and examined their associations with two health outcomes: functioning and self-reported health status. Across all approaches, the expected negative relationships were confirmed, with higher secondary health condition burden associated with poorer functioning and lower self-reported health. The scoring approach with higher but moderate correlation was the version that combined the modified SCI-SCS with mortality-based weights, although the unweighted modified SCI-SCS performed nearly as well.

Our findings highlight the potential of self-reported data to measure secondary health condition burden in populations where clinical data are limited, as is often the case in SCI research. Weighted scoring approaches are particularly valuable in complex conditions such as SCI, where multiple health problems interact and contribute to both clinical outcomes and quality of life. While previous studies have described the prevalence of secondary health conditions [3], reported causes of mortality [2, 25], or included comorbidity rates as control variables [2, 26], to our knowledge, no prior research has validated such scores against independent health outcomes. This study, therefore, provides an important step toward demonstrating that the SCI-SCS is more than a descriptive tool, and an outcome measure with potential for risk adjustment and comparative research.

Although these scoring approaches were developed specifically for individuals with an SCI, evaluating their applicability in diverse health conditions is a pertinent and timely endeavor. This study shows different ways to construct weighted scores using self-reported data and examines their relationships with other health indicators. In fact, the estimated scores performed well when compared to alternative health indicators, such as functioning or self-reported measures of health.

Country-level differences in correlation strength suggest that contextual factors—including healthcare system characteristics, access to services, rehabilitation practices, and cultural perceptions of health—may influence how secondary health conditions translate into functioning and perceived health [27]. Differences in prevalence, reporting practices, and treatment availability may further explain observed variability across countries [28]. Importantly, correlations tended to be more stable in larger national samples, underlining the importance of adequate sample sizes in cross-country comparisons.

Despite these findings, some limitations should be acknowledged. First, there is a 7-item overlap between the modified SCI-SCS scores and the functioning measure, which inevitably inflates the correlations. This shared content increases the associations by capturing part of the item overlap rather than exclusively reflecting the broader relationship between secondary health conditions and functioning. However, because all scores are equally affected, this limitation does not influence the relative differences observed among the scoring approaches. To address this issue, we included a second outcome—self-reported health status—which does not share item overlap with the modified SCI-SCS. The consistency of the results across both outcomes allows us to keep our conclusions regarding the performance of the weighted and unweighted scores. It is important to clarify that the functioning score used here is not intended as a directly comparable index but rather as a broad operationalization of health integrating not only the secondary health problems but also more items of body functions, activity, and participation domains and is derived using Item Response Theory methods [23]. Still, further validation using independent outcomes such as mortality or clinical indicators would provide an even stronger evidence base. Second, variability in data collection across countries may introduce self-selection bias and limit comparability [18]. Finally, incomplete reporting of some health conditions (e.g., diabetes, cancer) restricted the comprehensiveness of the index and highlights a need for more systematic data collection in future surveys.

## Conclusion

The modified SCI-SCS, as implemented in the InSCI survey, demonstrated moderate but consistent associations with functioning and self-reported health, supporting its utility as a proxy for overall health status in individuals with SCI. Incorporating mortality weights yielded marginal improvements, suggesting that while weighting can enhance measurement precision, the added complexity may not always be necessary.

This study contributes to advancing methods for assessing health burden in SCI by demonstrating feasible ways to construct weighted self-reported indices. Such approaches hold promise for both clinical research and international comparisons, where objective clinical data are often scarce. Future research should refine these scoring methods, test their predictive validity for long-term outcomes, and explore their applicability to other populations facing complex multimorbidity.

## Supplementary information

Apprendix

## Data Availability

The data that support the findings of this study are available from the International Spinal Cord Injury Community Survey (InSCI) Study Center, but restrictions apply to the availability of these data, which were used under license for the current study, and so are not publicly available. Data are however available from the authors upon reasonable request and with permission of the InSCI Study Center. To request the data please contact ana.ona@paraplegie.ch.
