# Supplementary material for: Evaluating the modified spinal cord injury secondary conditions scale (SCI-SCS) combining severity and mortality-based weights
Source: Spinal Cord. 2026 Jan 16;64(2):126–33. doi: 10.1038/s41393-026-01167-4 (PMC13661272; doi:10.1038/s41393-026-01167-4)
Supplement: Supplementary file 1 [file 41393_2026_1167_MOESM1_ESM.docx]

**Appendix**

**Table A1: Mortality weights for SCI secondary health conditions**

| **Condition** | **Weight** | **Justification** | **Reference** |
| --- | --- | --- | --- |
| Respiratory problems | 3 | Directly impacts vital functions, well-documented link to mortality | 32,33 |
| Circulatory problems | 3 | Directly impacts vital functions, well-documented link to mortality | 34,35 |
| Bowel dysfunction | 2 | Associated with complications affecting health, moderate mortality risk | 36 |
| Bladder dysfunction | 2 | Associated with complications affecting health, moderate mortality risk | 36,37 |
| Muscle spasms (spasticity) | 2 | Can contribute to complications such as pain and immobility | 36 |
| Pressure ulcers | 2 | Risk factor for infection and hospitalization | 37 |
| Sensation loss–related injuries | 2 | Can lead to infections or complications | 36 |
| Autonomic dysreflexia | 2 | Potentially life-threatening episodes, intermediate risk | 38 |
| Urinary tract infections | 2 | May lead to sepsis if untreated | 36,37 |
| Depression | 2 | Linked to poorer self-care and health outcomes | 36 |
| Sleep disturbances | 1 | Impacts quality of life but minimal direct mortality risk | 36 |
| Sexual dysfunction | 1 | Mainly affects quality of life | 36 |
| Contractures | 1 | Functional limitation, low mortality risk | 36 |
| Postural hypotension | 1 | Usually manageable, low mortality risk | 36 |
| Pain | 1 | Chronic pain impacts quality of life, not direct mortality | 36 |

**Table A2: Total Correlations**

| **Scores** |  | **Functioning** | |  | **Self-reported Health Status** | |
| --- | --- | --- | --- | --- | --- | --- |
|  |  | **Pearson coefficient** | **Confidence interval** |  | **Spearman coefficient** | **Confidence interval** |
| Score 1: modified SCI-SCS |  | -0.602 | [-0.615;-0.59] |  | -0.384 | [-0.4;-0.367] |
| Score 2 |  | -0.614 | [-0.626;-0.602] |  | -0.399 | [-0.415;-0.382] |
| Score 3 |  | -0.538 | [-0.552;-0.524] |  | -0.395 | [-0.355;-0.32] |
| Score 4 |  | -0.527 | [-0.541;-0.513] |  | -0.389 | [-0.351;-0.316] |
| Score 5 |  | -0.575 | [-0.588;-0.562] |  | -0.322 | [-0.34;-0.305] |
| Score 6 |  | -0.574 | [-0.586;-0.56] |  | -0.316 | [-0.333;-0.298] |
| Score 7 |  | -0.602 | [-0.614;-0.589] |  | -0.394 | [-0.411;-0.378] |
| Score 8 |  | -0.596 | [-0.608;-0.583] |  | -0.386 | [-0.402;-0.369] |

**Table A3: Total Correlation for Switzerland**

| **Scores** |  | **Functioning** | |  | **Self-reported Health Status** | |
| --- | --- | --- | --- | --- | --- | --- |
|  |  | **Pearson coefficient** | **Confidence interval** |  | **Spearman coefficient** | **Confidence interval** |
| Score 1: modified SCI-SCS |  | -0.545 | [-0.584;-0.503] |  | -0.429 | [-0.475;-0.381] |
| Score 2 |  | -0.575 | [-0.612;-0.535] |  | -0.440 | [-0.485;-0.392] |
| Score 3 |  | -0.448 | [-0.493;-0.401] |  | -0.435 | [-0.481;-0.388] |
| Score 4 |  | -0.441 | [-0.486;-0.394] |  | -0.420 | [-0.466;-0.371] |
| Score 5 |  | -0.526 | [-0.567;-0.484] |  | -0.340 | [-0.39;-0.288] |
| Score 6 |  | -0.535 | [-0.575;-0.493] |  | -0.331 | [-0.381;-0.278] |
| Score 7 |  | -0.542 | [-0.581;-0.5] |  | -0.435 | [-0.48;-0.387] |
| Score 8 |  | -0.548 | [-0.587;-0.506] |  | -0.417 | [-0.463;-0.368] |
